# Associations Between Peer Victimization and Aggression and Three Types of Domestic Violence in Adolescents with Attention-Deficit/Hyperactivity Disorder

**DOI:** 10.3390/children12040422

**Published:** 2025-03-28

**Authors:** Po-Chun Lin, Ching-Shu Tsai, Ray C. Hsiao, Cheng-Fang Yen

**Affiliations:** 1 Department of Psychiatry, E-Da Hospital, Kaohsiung 82445, Taiwan; bernie0421@gmail.com; 2 School of Medicine, College of Medicine, I-Shou University, Kaohsiung 82445, Taiwan; 3 Department of Child and Adolescent Psychiatry, Chang Gung Memorial Hospital, Kaohsiung Medical Center, Kaohsiung 83341, Taiwan; jingshu@cgmh.org.tw; 4 College of Medicine, Chang Gung University, Taoyuan City 33302, Taiwan; 5 Department of Psychiatry and Behavioral Sciences, School of Medicine, University of Washington, Seattle, WA 98195, USA; rhsiao@u.washington.edu; 6 Department of Psychiatry, Children’s Hospital and Regional Medical Center, Seattle, WA 98105, USA; 7 Department of Psychiatry, Kaohsiung Medical University Hospital, Kaohsiung 80708, Taiwan; 8 Department of Psychiatry, School of Medicine, College of Medicine, Kaohsiung Medical University, Kaohsiung 80708, Taiwan

**Keywords:** adolescents, aggression, attention-deficit/hyperactivity disorder, child-to-parent violence, parent-to-child aggression, peer victimization, witness to domestic violence

## Abstract

Background/Objectives: Peer victimization and aggression can be detrimental to the physical and mental health of adolescents. Adolescents with attention-deficit/hyperactivity disorder (ADHD) are one of the risk groups involved in peer victimization and aggression. The association between multiple types of domestic violence and engagement in peer victimization and aggression warrants more examination in adolescents with ADHD. This study examined the associations of parent-to-child aggression (PCA), witness to domestic violence (WDV), and child-to-parent violence (CPV) with the experiences of peer victimization and aggression among adolescents with a clinical diagnosis of ADHD in Taiwan. Materials and Methods: In total, 247 adolescents with ADHD (206 boys and 41 girls, mean age [*SD*] = 13.2 [2.0] years) agreed to participate in this study. Adolescents’ peer victimization and aggression, PCA, WDV, and CPV in the previous year were collected. Results: This study found that 28.3% and 12.6% of adolescents with ADHD reported experiences of peer victimization and aggression, respectively. The rates of having PCA, WDV, and CPV ranged from 38.1% to 56.3%. The results of multivariable logistic regression analysis found that child-to-parent financial demand (*p* = 0.016) and child-to-parent control or domination (*p* = 0.018) significantly correlated with the experiences of peer victimization. PCA (*p* = 0.010) and child-to-parent control or domination (*p* = 0.042) significantly correlated with the experiences of peer aggression. Conclusions: The results of this study show that both CPV and PCA significantly correlate with the experiences of peer victimization and aggression in adolescents with ADHD. CPV and PCA should be included in adolescent prevention programs.

## 1. Introduction

Both peer victimization and aggression put adolescents at risk for poor psychological outcomes such as mood disorders [1,2,3,4], anxiety [1,3,5], psychological harm [6], alcohol and substance use [7], psychosis [4], suicidal ideation and attempts [1,3,8], nonsuicidal self-injury [1], sleep problems [1,9], reduced health-related quality of life [1,10], peer rejection [11], and lower rates of graduation from high school [1]. The prevention and early detection of peer victimization and aggression are important health issues.

Attention-deficit/hyperactivity disorder (ADHD) is a common neurodevelopmental disorder [12]. The prevalence of ADHD in adolescents aged 12 to 18 years is 5.6% [13]. The core symptoms of ADHD include inattentiveness, hyperactivity, and impulsiveness [14]. A study on a nationally representative sample of 4816 children in Taiwan found that the prevalence of ADHD was 8.7% [15]. The prevalence in males was higher than that in females [16]. Adolescents with ADHD may have poor social relationships, low self-esteem, and poor academic performance [17]. A high proportion of adolescents with ADHD experience peer victimization and aggression [18,19,20,21,22]. For example, a study in the United States found that 57% of young adolescents with ADHD reported experiencing at least one victimization behavior once per week or more frequently, with higher rates of relational victimization (51%) than reputational victimization (17%) or physical victimization (14%) [21]. A study in Taiwan found that 20.2% and 14.0% of adolescents with ADHD reported peer victimization and aggression, respectively [18]. Compared to girls, boys were more likely to report peer victimization and aggression in Taiwan [23]. Younger students were more likely than older ones to report peer victimization and aggression in Taiwan [23]. Peer victimization and aggression are significantly associated with depression, anxiety, suicidality, poor sleep quality, pain, and low quality of life in adolescents with ADHD [21,24,25,26]. Effective prevention and intervention strategies for peer victimization and aggression require school and parent collaboration [27]. However, schools and parents often have difficulties communicating and working together to help children with ADHD [28]. Identifying the factors related to the experiences of peer victimization and aggression is important for developing intervention programs for bullying in adolescents with ADHD.

According to the general aggression model [29], both personal and environmental factors contribute to peer victimization and aggression in adolescents with ADHD. Studies have found that personal (e.g., male, young age, inattention, high behavioral inhibition, conduct problems, deficits in working memory, social skill deficits, no medication use, comorbid autism spectrum disorders, intellectual disability, learning disorders, and body image disorders), family (e.g., low satisfaction with family relationships and financial strain in the family), and peer and school (e.g., friendship difficulties and school problems) factors were associated with peer victimization and aggression among adolescents with ADHD [18,19,30,31,32,33]. An investigation of the factors related to peer victimization and aggression in adolescents with ADHD can help develop preventive and intervention strategies.

The question of whether domestic violence increases the risk of peer victimization and aggression has been of great interest to researchers in recent years. A review study demonstrated the high prevalence of aggression and victimization among children with ADHD; a high proportion of them suffer from polyvictimization [34]. Compared to children without ADHD, children with ADHD were 2.62 times more likely to experience four or more types of victimization [35]. A study on a large community sample (*N* = 76,227) found that children with ADHD had a higher prevalence of witnessing domestic violence and neighborhood violence compared to children without ADHD [36]. It has been hypothesized that altered neurocognitive functioning following the victimization of domestic violence may shed light on why affected children are more likely to be victimized by their peers [37]. However, the experience of multiple types of victimization among children with ADHD is a highly under-researched area [34]. The associations of the experiences of peer victimization and aggression with multiple types of domestic violence in adolescents with ADHD warrant further study.

Parent-to-child aggression (PCA), witness to domestic violence (WDV), and child-to-parent violence (CPV) are three types of domestic violence that adolescents may experience [38]. A prospective study in the United States found that PCA predicted child externalizing and internalizing behaviors and social and scholastic competence problems [38]. A study in Japan found that the experiences of peer victimization and aggression were higher among adolescents with PCA than those without PCA [39]. Children with WDV had significantly worse psychosocial outcomes relative to non-witnesses [40]. WDV also increased the risk of peer victimization among adolescents [41]. CPV can be a precursor to various forms of violent crime [42]. Studies have found a significant association between CPV and peer victimization and aggression in adolescents [43,44]. Adolescents with ADHD are a high-risk group for experiencing both peer victimization and aggression and domestic violence [18,19,20,36]. However, the associations of PCA, WDV, and CPV with peer victimization and aggression have not been examined yet.

This study examined the associations of PCA, WDV, and CPV with peer victimization and aggression in adolescents with a clinical diagnosis of ADHD. We hypothesized that PCA, WDV, and CPV are significantly associated with peer victimization and aggression in adolescents with ADHD.

## 2. Materials and Methods

### 2.1. Participants

In this cross-sectional questionnaire survey study, we distributed surveys to adolescents with ADHD. Adolescents with ADHD from six child psychiatry outpatient clinics at two hospitals in Taiwan were included for analysis. Adolescents with ADHD meeting the following criteria were included in this study: (1) being 11–18 years of age and (2) having received a diagnosis of ADHD by a certified child psychiatrist in accordance with the *Diagnostic and Statistical Manual of Mental Disorders, Fifth Edition* [12]. Adolescents who had the comorbidity of intellectual disability, severe autism spectrum disorder, bipolar disorder, schizophrenia, or any other cognitive deficits that may impede their understanding of this study’s purposes and completion of the research questionnaire were excluded.

Three child psychiatrists reviewed the medical records of adolescents with ADHD who visited the selected outpatient clinics between August 2023 and July 2024. A total of 259 adolescents with ADHD were consecutively approached. The child psychiatrists interviewed the adolescents and excluded 12 adolescents with ADHD because they had either a comorbid autism spectrum disorder (*n* = 6) or an intellectual disability (*n* = 6). Subsequently, the child psychiatrists explained this study’s purposes and procedures to the remaining adolescents and invited them to participate in this study. All participants were assured that their responses would remain confidential and that their participation or nonparticipation would not influence their right to receive medical services. In total, 247 adolescents with ADHD agreed to participate in this study. According to Hsieh et al. [45], the required sample size is 242, with a two-sided significance level of 0.05 and a power of 0.80.

### 2.2. Procedures

When inviting adolescents and parents to participate in this study, the researcher explained the purpose of this study to them, ensuring the confidentiality of the questionnaire results and their medical rights. The adolescents were told that the questionnaire was about their experiences with their families and school peers. The results of the questionnaires were classified by numbers instead of their names, and the medical staff was not informed about the results of the answers they filled in. Participants filled out the questionnaire in the research room. The research questionnaire was reviewed by experts to confirm that the adolescents were able to understand the meaning of the questions. If participants had any questions about the content of the questionnaire, they could ask the research assistant at any time. If participants had any ideas to discuss with the psychiatrists after completing the questionnaire, they were welcome to do so.

### 2.3. Ethical Considerations

The protocol of the present study was approved by the Institutional Review Boards of the Kaohsiung Medical University Hospital (KMUHIRB-SV(II)-20210113, date: 28 March 2023) and the Chang Gung Memorial Hospital, Kaohsiung Medical Center (202102157A3C601, date: 13 April 2023). Informed consent was obtained from all adolescents and their parents. This study employed a survey design and did not involve experiments on humans or human tissue samples. This study was conducted in accordance with the Declaration of Helsinki and the Recommendations for the Conduct, Reporting, Editing, and Publication of Scholarly Work in Medical Journals by the International Committee of Medical Journal Editors.

### 2.4. Measures

#### 2.4.1. Peer Victimization and Aggression

The self-reported Chinese version of the School Bullying Experience Questionnaire (C-SBEQ) was used to evaluate participants’ experiences of peer victimization and aggression at schools and cram schools in the previous year [46,47]. Eight items were used to assess the experiences of peer victimization through social (e.g., “Other students do not talk to you or answer you?”), verbal (e.g., “Other students speak ill of you?”), and physical bullying (e.g., “Are you beaten up?”), and another eight items were used to assess the experiences of peer aggression (e.g., “Do you call other students names?”). Each item was answered on a 4-point Likert scale with endpoints of 0 (*never*) to 3 (*all the time*). The C-SBEQ has acceptable reliability and validity [27]. In this study, the Cronbach’s α values were 0.76 and 0.72 for peer victimization and aggression, respectively. Participants who answered 2 or 3 on any item among the items assessing peer victimization and the items assessing peer aggression were identified to have the experiences of peer victimization and aggression, respectively.

#### 2.4.2. Child-to-Parent Violence

We used the 14-item adolescent-reported Chinese version [48] of the Child-to-Parent Violence Questionnaire (CPV-Q) [49] to evaluate four types of CPV in the year preceding evaluation, including psychological aggression (four items, e.g., “I have told my parents ‘I hate you!’ and ‘I wish you were dead’”), physical aggression (three items, e.g., “I have thrown things at my parents”), financial demand (three items, e.g., “I have demanded that my parents buy me things I know they cannot afford”), and control and domination (four items, e.g., “I have told my parents that at home they have to do what I want”). Each item was rated on a 5-point scale ranging from 0 (*never*) to 4 (*six times or more*). The original and Chinese versions of CPV-Q have acceptable reliability and validity [48,49]. In the present study, the internal consistency (McDonald’s ω) of the four domains of both CPV-Q versions ranged from 0.65 to 0.78. Adolescents whose answers were not 0 for any item in the child version of the CPV-Q were regarded as having CPV.

#### 2.4.3. Parent-to-Child Aggression

We adopted seven items from the CPV-Q to develop the Parent-to-Child Violence Questionnaire (PCV-Q) for evaluating parental verbal (four items, e.g., “My father or mother has made negative, offensive, and insulting comments about me”) and physical violence (three items, e.g., “My father or mother has kicked, slapped and punched me”) against adolescents in the year preceding evaluation. The items in the PCV-Q were rated on the same 5-point scale as that used in the CPV-Q. The internal consistency (McDonald’s ω) of the PCV-Q was calculated as 0.76. Answers other than 0 for any item of the PCV-Q were indicative of PCA.

#### 2.4.4. Witness to Domestic Violence

We adopted seven items from the CPV-Q to develop the Witness to Domestic Violence Questionnaire (WDA-Q) for evaluating adolescents who witness verbal (e.g., “Family members used to insult each other”) and physical violence (e.g., “Family members have kicked, slapped and punched each other”) among adult family members in the year preceding the evaluation. The items in the WDA-Q were rated on the same 5-point scale as that used in the CPV-Q. The internal consistency (McDonald’s ω) of the WDA-Q was calculated as 0.72. Answers other than 0 for any item of the WDA-Q were indicative of adolescents witnessing violence among adult family members.

#### 2.4.5. ADHD Symptoms

We used the parent-reported domain of ADHD symptoms (7 items) in the Chinese version of the Child Behavior Checklist for Ages 6–18 to evaluate adolescents’ ADHD symptoms [50,51]. We also used the recommended T-score transformations of raw scores, which were adjusted for age and sex differences in ADHD symptoms found in normative samples. A higher total score indicates more severe ADHD symptoms. This checklist has an internal consistency (Cronbach’s α) of 0.55–0.90 and a 1-month test–retest reliability (Pearson’s *r*) of 0.51–0.74, along with high construct validity (eight-factor structure) [52,53].

### 2.5. Data Analysis

All statistical analyses were conducted using IBM SPSS Statistics version 24.0 (IBM, Armonk, NY, USA). Descriptive statistics (presented as means and frequencies) were used to summarize the study sample’s characteristics. A bivariable logistic regression analysis was conducted to examine the associations of demographic characteristics, ADHD symptoms, and three types of domestic violence with the experiences of peer victimization and aggression. Factors that exhibited a significant correlation with peer victimization and aggression in the bivariable logistic regression analysis were included in a multivariable logistic regression analysis to further explore their associations with peer victimization and aggression. The results are presented as odds ratios (ORs) and 95% confidence interval (CI). A *p*-value less than 0.05 was considered statistically significant.

## 3. Results

### 3.1. Rates of Peer Victimization and Aggression and Domestic Violence

Table 1 presents the participants’ demographic characteristics, ADHD symptoms, PCA, WDV, CPV, and peer victimization and aggression. Most of the participants (83.4%) were boys, and the mean age of the participants was 13.2 years (*SD* = 2.0 years). The mean score of ADHD symptoms on the CBCL/6–18 was 61.9 (*SD* = 7.7). In total, 28.3% and 12.6% of adolescents reported having experiences of peer victimization and aggression, respectively. Regarding the experiences of domestic violence, 38.9% experienced PCA; 41.3% had WDV; 38.1% had child-to-parent psychological aggression; 7.7% had child-to-parent physical aggression; 26.7% had child-to-parent financial demand; 38.1% had child-to-parent control or domination; and 56.3% had at least one type of CPV. The mean number of experiences of victimization, including parent-to-child aggression, witness to domestic violence, and peer victimization, was 0.7 (*SD* = 0.8); 39 participants (15.8%) reported two types of victimization, and 5 participants (2.0%) reported three types of victimization.

### 3.2. Factors Related to Peer Victimization and Aggression: Bivariable Logistic Regression

Table 2 presents the factors related to the experiences of peer victimization and aggression examined by bivariable logistic regression. The severity of ADHD symptoms (*p* = 0.002), PCA (*p* = 0.025), child-to-parent psychological aggression (*p* = 0.033), child-to-parent financial demand (*p* = 0.009), and child-to-parent control or domination (*p* = 0.001) were significantly correlated with the experiences of peer victimization. PCA (*p* = 0.006), child-to-parent psychological aggression (*p* = 0.006), and child-to-parent control or domination (*p* = 0.017) were significantly correlated with the experiences of peer aggression. The severity of ADHD symptoms was mildly but significantly correlated with peer aggression (OR = 1.050, 95% CI: 1.001, 1.101, *p* = 0.046).

### 3.3. Factors Related to Peer Victimization and Aggression: Multivariable Logistic Regression

Factors that exhibited a significant correlation with peer victimization and aggression in the bivariable logistic regression analysis were included in a multivariable logistic regression analysis (Table 3). Child-to-parent financial demand (*p* = 0.016) and child-to-parent control or domination (*p* = 0.018) were significantly correlated with the experiences of peer victimization. PCA (*p* = 0.010) and child-to-parent control or domination (*p* = 0.042) were significantly correlated with the experiences of peer aggression.

## 4. Discussion

The present study found that 28.3% and 12.6% of adolescents with ADHD reported having experiences of peer victimization and aggression, respectively. The rates of having PCA, WDV, and CPV ranged from 38.1% to 56.3%. The results of this study indicated that a high proportion of adolescents with ADHD experienced various types of aggression at home and from peers. Intervention programs for adolescent-involved aggression must comprehensively assess the aggression that occurs at home and in peer groups.

The present study found that PCA significantly correlated with the experiences of peer aggression in adolescents with ADHD. Although the cross-sectional study design limited the inference of the temporal relationship between PCA and peer aggression, the results of previous studies have proposed some potential mechanisms that account for the association between PCA and peer aggression. First, studies have found that PCA is significantly and positively related to children’s rejection sensitivity [54]. Individuals who have high sensitivity to social rejection may anxiously expect, perceive, and overreact to social rejection, thus increasing the risk of aggression [55]. A prospective study on 3525 adolescents in China found that the association between childhood emotional abuse and peer aggression was mediated by rejection sensitivity [56]. Second, a review study identified a significant association between PCA and mental health problems in children [57]. Mental health problems such as depression can further exacerbate rejection sensitivity and increase the risk of peer aggression [58]. In addition, several hypothesized mechanisms may account for the correlation between PCA and the experiences of peer aggression in adolescents with ADHD and warrant examination. First, genetic and biological makeup may create a predisposition for aggressive behavior. For example, genetic variations in proteins regulating the synthesis, degradation, and transport of serotonin and dopamine mediate behavioral variability observed in aggression [59]. Second, through negative parenting practices, such as corporal punishment, children may learn that such treatment can be used to control others, initiating an intergenerational transmission of aggression [60]. Third, impulsivity is one of the core symptoms of ADHD [12]. Impulsivity may increase the likelihood of adolescents’ fierce resistance to parental discipline, which, in turn, may increase parent–child conflict [61]. Impulsivity also increases the risk of peer aggression in adolescents with ADHD [62]. Fourth, PCA may be an inappropriate parental response to a child’s peer aggressive behavior. However, these hypothesized mechanisms warrant further study.

The present study found that child-to-parent control/domination significantly correlated with the risk of peer aggression in adolescents with ADHD. Child-to-parent control/domination is reflected in behaviors such as making unrealistic demands on parents (for example, “I have demanded my parents to stop what they are doing to pay attention to me”) or controlling the way the household is run (for example, “I have told my parents that at home they have to do what I want”) [49]. Child-to-parent control/domination demonstrates adolescents’ attitudes toward the expectation of playing a dominant role in interpersonal interactions. Such an attitude may extend from the treatment of parents at home to peer interactions outside the home, resulting in aggressive behavior toward peers. The present study also found that child-to-parent control/domination was significantly correlated with the risk of peer victimization in adolescents with ADHD. Adolescents may be ostracized by their peers and suffer from peer victimization because they expect to take the lead in interpersonal interactions. Alternatively, adolescents may experience peer victimization outside the home and display control/domination toward parents at home to gain compensatory outlet. Further study is needed to test whether these hypothesized mechanisms account for the associations of child-to-parent control/domination with the experiences of peer victimization and aggression in adolescents with ADHD.

The present study found that child-to-parent financial demand was significantly correlated with peer victimization in adolescents with ADHD. Child-to-parent financial demand includes behaviors such as stealing money or parents’ belongings, demanding that their parents buy things they feel they cannot afford, or incurring debts the parents must cover [49]. It is possible that an adolescent may suffer financial loss or be threatened with money in peer victimization and thus exert financial violence on their parents at home. Although the present study did not find a significant association between WDV and peer victimization and aggression in adolescents with ADHD, WDV has a negative influence on adolescents’ mental health and thus warrants prevention and early intervention [40].

### 4.1. Implications

Based on the findings of this study, we make the following suggestions. First, a high proportion of adolescents with ADHD experience peer victimization and aggression. Given that peer victimization and aggression can compromise physical and mental health in adolescents with ADHD [24,25,26], intervention programs are needed for the prevention and early detection of peer victimization and aggression in this group. Second, interventions that protect people with mental disabilities from violence toward those with various forms of disabilities focus on training and setting up channels and facilities for victims to seek help [63]. However, the prevention and treatment of peer victimization and aggression in adolescents with ADHD should be also approached from an ecological systems perspective [64]. Health professionals need to regularly evaluate not only the experiences of peer victimization and aggression but also PCA and CPV in adolescents with ADHD and explore the relationships among them. Schools and parents need to work together to eliminate the multifaceted crisis of violence among adolescents with ADHD. Third, health professionals should help parents of adolescents with ADHD to develop parenting skills to reduce PVA and enhance adolescents’ motivation to discuss their school lives with their parents. Fourth, parents should be aware of the associations of child-to-parent control/domination and financial demand with peer victimization and aggression. Parents of adolescents with ADHD should be also taught how to manage adolescents’ child-to-parent control/domination and financial demand.

### 4.2. Limitations

This study has several limitations. First, this study did not invite a group of adolescents without ADHD as the comparative group. Further studies are needed to examine the associations of the risk factors identified in this study with the experiences of peer victimization and aggression in adolescents without ADHD. Second, adolescents with ADHD were recruited from outpatient clinics, where they were actively receiving pharmacological or psychological therapy and thus had mild ADHD symptomatology (mean score of ADHD symptoms on the CBCL/6–18 = 61.9). However, even though the ADHD symptoms were quite mild in the participants of this study, ADHD symptoms were still mildly and significantly associated with peer aggression, indicating that adolescents with ADHD involved in peer aggression deserve attention. Future studies should investigate whether our findings can be extended to adolescents with ADHD who are not receiving medical treatment. Third, the temporal associations between domestic violence and peer victimization and aggression could not be determined because of the cross-sectional design of this study. Fourth, both domestic violence and peer victimization and aggression were collected from adolescents; there might be single-rater and recall biases. Fifth, although we proposed several possible mechanisms accounting for the association of domestic violence with peer victimization and aggression, the mechanisms warrant further study. Sixth, this study did not examine the intention to cause harm and power imbalance between adolescents who experienced peer victimization and aggression.

## 5. Conclusions

Our results indicate that a high proportion of adolescents with ADHD are involved in peer victimization and aggression. Child-to-parent control/domination and financial demand and PCA were significantly correlated with peer victimization and aggression in adolescents with ADHD. Intervention programs for peer victimization and aggression in adolescents with ADHD should include CPV and PCA as risk factors. Health professionals should help parents of adolescents with ADHD develop parenting skills to reduce PVA, manage CPV, and help adolescents manage peer victimization and aggression.

## Figures and Tables

**Table 1:** Demographic characteristics, ADHD symptoms, parent-to-child aggression, witness to domestic violence, child-to-parent violence, and peer victimization and aggression (*N* = 247).

Variables	*n* (%)	Mean (SD)	Range
Adolescent’s gender			
Girl	41 (16.6)		
Boy	206 (83.4)		
Adolescent’s age (years)		13.2 (2.0)	11–18
ADHD symptoms on the CBCL/6–18		61.9 (7.7)	50–80
Parent-to-child aggression	96 (38.9)		
Witness to domestic violence	102 (41.3)		
Child-to-parent psychological aggression	94 (38.1)		
Child-to-parent physical aggression	19 (7.7)		
Child-to-parent financial demand	66 (26.7)		
Child-to-parent control or domination	94 (38.1)		
Any child-to-parent aggression	139 (56.3)		
Having peer victimization	70 (28.3)		
Having peer aggression	31 (12.6)		
Number of victimization experiences ^a^			
0	111 (44.9)		
1	92 (37.2)		
2	39 (15.8)		
3	5 (2.0)		

^a^: Total number of experiences of victimization, including parent-to-child aggression, witness to domestic violence, and peer victimization. ADHD: attention-deficit/hyperactivity disorder; CBCL/6–18: Child Behavior Checklist for Ages 6–18.

**Table 2:** Factors related to the experiences of peer victimization and aggression: bivariable logistic regression.

Variables	Peer Victimization		Peer Aggression	
	OR (95% CI)	*p*	OR (95% CI)	*p*
Adolescent’s age	0.877 (0.759, 1.013)	0.074	0.959 (0.793, 1.160)	0.668
Adolescent’s sex	0.555 (0.276, 1.118)	0.099	1.040 (0.374, 2.889)	0.940
ADHD symptoms	1.060 (1.022, 1.099)	0.002 **	1.050 (1.001, 1.101)	0.046 *
Parent-to-child violence	1.902 (1.084, 3.335)	0.025 *	3.948 (1.768, 8.817)	0.001 **
Witness to domestic violence	1.287 (0.736, 2.250)	0.376	0.755 (0.345, 1.654)	0.483
Child-to-parent psychological aggression	1.842 (1.049, 3.233)	0.033 *	2.977 (1.371, 6.462)	0.006 **
Child-to-parent physical aggression	1.183 (0.431, 3.246)	0.745	1.339 (0.367, 4.890)	0.658
Child-to-parent financial demand	2.222 (1.221, 4.042)	0.009 **	1.141 (0.496, 2.623)	0.756
Child-to-parent control or domination	2.566 (1.455, 4.523)	0.001 **	2.551 (1.186, 5.487)	0.017 *

ADHD: attention-deficit/hyperactivity disorder; CI: confidence interval; OR: odds ratio. *: *p* < 0.05; **: *p* < 0.01.

**Table 3:** Factors related to the experiences of peer victimization and aggression: multivariable logistic regression.

	Peer Victimization		Peer Aggression	
	OR (95% CI)	*p*	OR (95% CI)	*p*
ADHD symptoms	1.033 (0.993, 1.075)	0.105	1.017 (0.967, 1.069)	0.510
Parent-to-child violence	1.292 (0.685, 2.436)	0.429	2.992 (1.302, 6.880)	0.010 *
Child-to-parent psychological aggression	1.021 (0.521, 2.002)	0.951	–	–
Child-to-parent financial demand	2.078 (1.148, 3.760)	0.016 *	–	–
Child-to-parent control or domination	2.248 (1.152, 4.386)	0.018 *	3.209 (1.044, 9.863)	0.042 *

ADHD: attention-deficit/hyperactivity disorder; CI: confidence interval; OR: odds ratio. *: *p* < 0.05.

## Data Availability

The data are available upon reasonable request to the corresponding authors due to the rule of institutional review boards.
